# Rapid analysis of heterogeneously methylated DNA using digital methylation-sensitive high resolution melting: application to the *CDKN2B *(*p15*) gene

**DOI:** 10.1186/1756-8935-1-7

**Published:** 2008-11-03

**Authors:** Ida LM Candiloro, Thomas Mikeska, Peter Hokland, Alexander Dobrovic

**Affiliations:** 1 Molecular Pathology Research and Development Laboratory, Department of Pathology, Peter MacCallum Cancer Centre, Melbourne, Victoria 8006, Australia; 2 Department of Haematology, Aarhus University Hospital, Aarhus, Denmark; 3 Department of Pathology, University of Melbourne, Parkville, Victoria 3010, Australia

## Abstract

**Background:**

Methylation-sensitive high resolution melting (MS-HRM) methodology is able to recognise heterogeneously methylated sequences by their characteristic melting profiles. To further analyse heterogeneously methylated sequences, we adopted a digital approach to MS-HRM (dMS-HRM) that involves the amplification of single templates after limiting dilution to quantify and to determine the degree of methylation. We used this approach to study methylation of the *CDKN2B *(*p15*) cell cycle progression inhibitor gene which is inactivated by DNA methylation in haematological malignancies of the myeloid lineage. Its promoter region usually shows heterogeneous methylation and is only rarely fully methylated. The methylation status of *CDKN2B *can be used as a biomarker of response to treatment. Therefore the accurate characterisation of its methylation is desirable.

**Results:**

MS-HRM was used to assess *CDKN2B *methylation in acute myeloid leukaemia (AML) samples. All the AML samples that were methylated at the *CDKN2B *promoter (40/93) showed varying degrees of heterogeneous methylation. Six representative samples were selected for further study. dMS-HRM was used to simultaneously count the methylated alleles and assess the degree of methylation. Direct sequencing of selected dMS-HRM products was used to determine the exact DNA methylation pattern and confirmed the degree of methylation estimated by dMS-HRM.

**Conclusion:**

dMS-HRM is a powerful technique for the analysis of methylation in *CDKN2B *and other heterogeneously methylated genes. It eliminates both PCR and cloning bias towards either methylated or unmethylated DNA. Potentially complex information is simplified into a digital output, allowing counting of methylated and unmethylated alleles and providing an overall picture of methylation at the given locus. Downstream sequencing is minimised as dMS-HRM acts as a screen to select only methylated clones for further analysis.

## Background

Epigenetic mechanisms, in particular DNA methylation, play a major role in the modulation of gene activity in cancer. Methylation has been shown to silence a large number of genes in nearly every type of cancer [1,2]. Whilst it is generally accepted that methylation of a promoter may be necessary for gene silencing, it is clear that in many cancers promoters are often heterogeneously methylated. This is an important issue both for the detection and quantitation of methylation.

The tumour suppressor gene *CDKN2B *(*p15*), which is silenced in a variety of haematological malignancies [3], is one such heterogeneously methylated locus. Silencing of *CDKN2B *expression can occur with only partial methylation of the promoter and many differently methylated *CDKN2B *alleles frequently co-exist [4-6].

Methylation of *CDKN2B *has been used as a biomarker for myelodysplastic syndrome and acute myeloid leukaemia (AML) [7], and as a prognostic indicator either alone or in combination with other loci [8,9]. Monitoring changes in *CDKN2B *methylation over time would also prove useful in assessing residual disease. However, due to its heterogeneity, quantification of *CDKN2B *methylation is challenging.

Methylation-sensitive high resolution melting (MS-HRM) is a methodology that is particularly suitable for the rapid analysis of clinical samples [10]. MS-HRM differentiates methylated and unmethylated templates on the basis of the marked difference in melting behaviour due to their different base compositions following bisulphite conversion. We have used MS-HRM to analyse methylated promoter regions in cancer [10,11], and the *H19/IGF2 *imprinting centre in imprinting disorders [12]. Here, we show that MS-HRM is an appropriate methodology for the detection of heterogeneously methylated cancer samples using the *CDKN2B *gene as an example.

Digital methylation-sensitive high resolution melting (dMS-HRM) was introduced as a methodology for counting methylated and unmethylated alleles of the *BRCA1 *gene [11]. In that case, dMS-HRM was used to confirm the MS-HRM analysis. In this communication, we show that dMS-HRM enables detailed analysis of DNA methylation in complex heterogeneously methylated templates, eliminating the need for sequencing analysis in most cases. This can be done in a time- and cost-effective fashion as we have shown using the clinically important *CDKN2B *gene as an example.

## Methods

### DNA samples

AML samples were obtained from patients referred to the Department of Haematology, Aarhus University Hospital, Denmark. DNA was re-dissolved in TE buffer (1×) at a final concentration of 5 ng/μl. Genomic DNA was extracted from peripheral blood of healthy controls using the QIAamp DNA Blood Mini Kit (Qiagen, Hilden, Germany) according to the manufacturer's instructions. The investigation was approved by the Peter MacCallum Cancer Centre Ethics of Human Research Committee (Approval number 02/26). Whole genome amplification (WGA) was performed as described previously [13]. The purpose of using DNA subjected to two rounds of WGA is to ensure that the DNA is completely unmethylated, as normal, healthy individuals may have low-level methylation at the *CDKN2B *promoter in peripheral blood cells [6].

### Bisulphite modification

200 ng of genomic DNA from the AML samples was subjected to bisulphite modification by using the EpiTect Bisulfite Kit (Qiagen) according to the manufacturer's instructions. DNA was eluted once in 20 μl of buffer EB. Universal Methylated DNA (Chemicon, Millipore, Billerica, MA) and WGA product were used as the controls. 500 ng of each was modified. The modified control DNA underwent a second elution in 30 μl of buffer EB.

### MS-HRM

PCR cycling and MS-HRM was performed on the Rotor-Gene 6000 (Corbett Research, Sydney, Australia), an HRM-enabled real time PCR instrument. Each sample was analysed in duplicate for MS-HRM.

Primers were designed according to the principles outlined in Wojdacz et al [14]. Briefly, the primers should contain a limited number of CpG dinucleotides (usually one or two) which should be kept as far as possible from the 3' end of the primers. This allows the control of PCR bias by appropriate choice of annealing temperature.

The primers used to amplify bisulphite-treated DNA were CDKN2B-F, 5'-GTTAGGCGTTTTTTTTTAGAAGTAATTTAGG-3' and CDKN2B-R, 5'-TACGACTTAAAACCCCGTACAATAACC-3' and do not amplify unmodified genomic DNA (data not shown). The amplified region corresponds to [GenBank: AL449423] nucleotides 99845 to 99958, and encompasses nine CpG dinucleotides. PCR was performed in 100 μl PCR tubes (Corbett Research) with a final volume of 20 μl, containing 200 nmol/l of each primer, 200 μmol/l of each dNTP, 0.5 U of HotStarTaq DNA polymerase (Qiagen) in the supplied PCR buffer containing 2.5 mmol/l MgCl_2_, 5 μmol/l SYTO9 (Invitrogen, Carlsbad, CA), and 10 ng of bisulphite-treated DNA. The initial denaturation (95°C, 15 minutes) was followed by 50 cycles for MS-HRM of 10 seconds at 95°C, 30 seconds at 59°C, 30 seconds at 72°C; one cycle of 1 minute at 95°C, 72°C for 1.5 minutes and a HRM step from 65°C to 90°C rising at 0.2°C per second, and holding for 1 second after each stepwise increment. The annealing temperature of 59°C was chosen as it gave a near-proportional amplification of methylated and unmethylated templates.

### Digital MS-HRM

The basis of dMS-HRM is the PCR amplification of single molecules following limiting dilution [15,16]. To perform limiting dilution, the DNA concentration is determined. Depending on the starting concentration, a dilution series (in the order of 1:1000 to 1:2000 for 10 ng/μl) should be used. The dilution that performs best (that is, a substantial proportion of negative reactions) is chosen and used for the final experimental set-up.

The Poisson distribution is used as a guideline to determine the expected distribution of templates. For example, if the sample is diluted such that one amplifiable template is found on average per reaction, 36.8% of reactions will have no templates, 36.8% will have one template, and 26.4% will have more than one template. Amplification will occur when one or more template molecules are present in the PCR reaction.

Amplifications from single templates can be readily identified when the melting curves are analysed. They show a smooth and sharp single signal. Melting curves from two (or more) templates generally result in signals showing two peaks if heteroduplexes are not formed, or more complex patterns when heteroduplexes are formed. Digital MS-HRM was performed using 60 cycles of amplification. Sixty replicates of diluted template were analysed per sample.

### Direct sequencing

dMS-HRM products were cleaned up with the PCR-M clean-up kit (Viogene, Taipei, Taiwan), according to the manufacturer's instructions, further processed with ExoSapIT (GE Healthcare, Little Chalfont, England), followed by the sequencing reaction using Big Dye Terminator v3.1 chemistry (Applied Biosystems, Foster City, CA) according to the manufacturer's instructions. Sequencing was performed in both directions using the PCR primers given above as sequencing primers. The initial denaturation (95°C, 1 minute) was followed by 30 cycles of 10 seconds at 95°C, 30 seconds at 59°C and 3 minutes at 72°C. The sequencing products were purified by ethanol precipitation and separated on a 3100 Genetic Analyser (Applied Biosystems). The sequencing data for the dMS-HRM products were analysed using BiQ Analyzer software (Max-Planck-Institut für Informatik, Saarbrücken, Germany) [17].

## Results

### MS-HRM of AML samples

**Figure 1:**
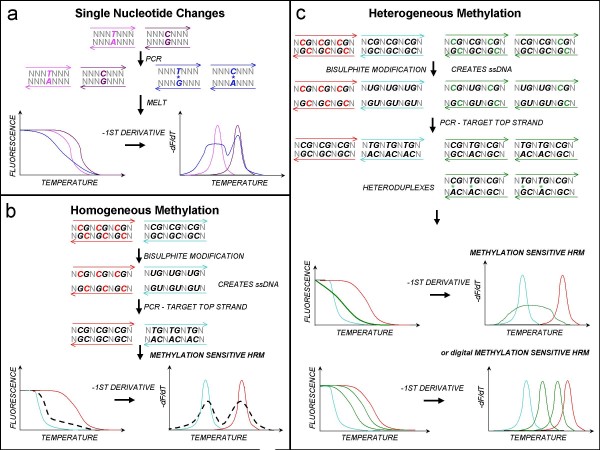
**High resolution melting: application to DNA methylation analysis.     **High resolution melting (HRM) tracks melting of PCR amplicons using an intercalating fluorescent dye. Amplicons with different sequences display different melting profiles, allowing identification of sequence variants. The panels show model sequences and then representations of the resultant normalised melting curves and Tm curves (negative first derivative of the melting curves). **Panel a: Single base changes**. HRM can distinguish heterozygotes from homozygotes due to formation of heteroduplexes (shown in blue). As heteroduplexes are less stable than homoduplexes (pink and purple), they will melt earlier. **Panel b: Homogeneous methylation**. Detection of methylated cytosines via HRM (MS-HRM) relies upon sequence changes introduced by bisulphite modification. Unmethylated cytosines (black Cs) are converted to uracils (Us), while methylated cytosines (red Cs) are resistant to modification. Only one strand is amplified. When a mixture of fully methylated and unmethylated templates are analysed, heteroduplexes are not formed if there are four or more CpG sites in the amplicon. **Panel c: Heterogeneous methylation**. When methylation is heterogeneous, heteroduplexes form because of the presence of molecules that differ only by a few bases. The large number of potential heteroduplexes leads to complex melting patterns. The original templates can be identified by digital analysis.

HRM differentiates between DNA molecules based on their sequence-dependent thermostability (Figure 1, panel a). MS-HRM takes advantage of extensive sequence changes introduced during bisulphite conversion of DNA to distinguish between methylated and unmethylated DNA (Figure 1, panel b). Amplicons derived from fully methylated sequences will not form heteroduplexes with amplicons derived from fully unmethylated sequences. In heterogeneously methylated populations in which molecules that differ by one, two or three bases are present, heteroduplexes will form with characteristic earlier melting due to the mismatches. Heterogeneously methylated samples result in extensive heteroduplex formation giving a complex profile (Figure 1, panel c).

**Figure 2:**
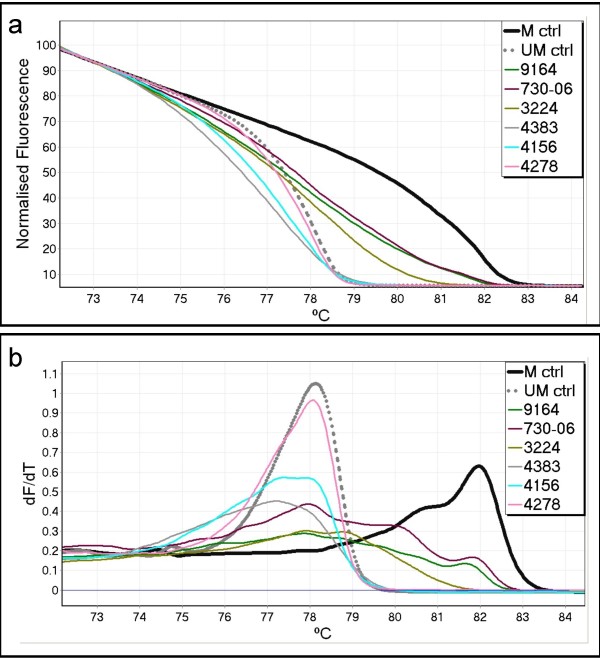
**Acute myeloid leukaemia samples analysed using conventional methylation-sensitive high resolution melting**. Methylation-sensitive high resolution melting can distinguish homogeneous from heterogeneous DNA methylation as each show characteristic melting profiles. The methylated DNA control is indicated by a solid black line (M ctrl), and the unmethylated whole genome amplification control by a broken grey line (UM ctrl). Six acute myeloid leukaemia (AML) samples are indicated by unbroken coloured lines. (a) shows the normalised melting curves. (b) shows the negative first derivative (or Tm) curves. It can be clearly seen that apart from sample 4278 which is unmethylated, none of the other five AML samples resemble the controls. In the Tm plots, these AML samples show broad melting regions which would be expected as a consequence of extensive heteroduplex formation. They begin melting before the unmethylated control, with three samples (3224, 9164, 730-06) continuing to melt in the region indicative of methylation. It should be noted that the methylated DNA control is not fully methylated and contains a typical left-shifted tail (see text).

Forty of the 93 acute myeloid leukaemia (AML) samples were methylated. All showed a heterogeneous melting profile. Figure 2 shows typical MS-HRM melting profiles of the *CDKN2B *promoter region in AML samples. Figure 2a shows the normalised melting pattern and Figure 2b shows the melting profile (negative first derivative of the raw melting pattern). The amplicons derived from methylated control DNA and the unmethylated WGA product define the range of methylation. The commercial methylated control shows a broad peak as it was substantially but not fully methylated (results not shown).

### dMS-HRM of AML samples

**Figure 3:**
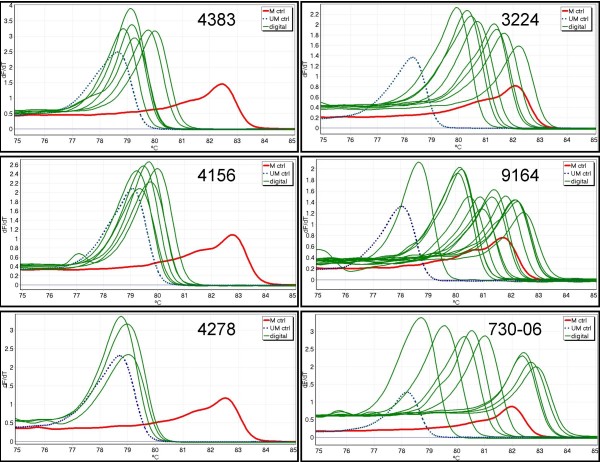
**dMS-HRM profiles of the six AML samples analysed in detail**. Negative first derivative (or Tm) curves are shown. The methylated control is displayed as a solid red line (M ctrl), and the unmethylated whole genome amplification control is shown as a dotted blue line (UM ctrl). The peaks from the digitally obtained amplicons are shown in green, while amplicons arising from multiple templates are omitted for clarity. Replicates showing identical peaks to those already displayed are also omitted for clarity.

**Figure 4:**
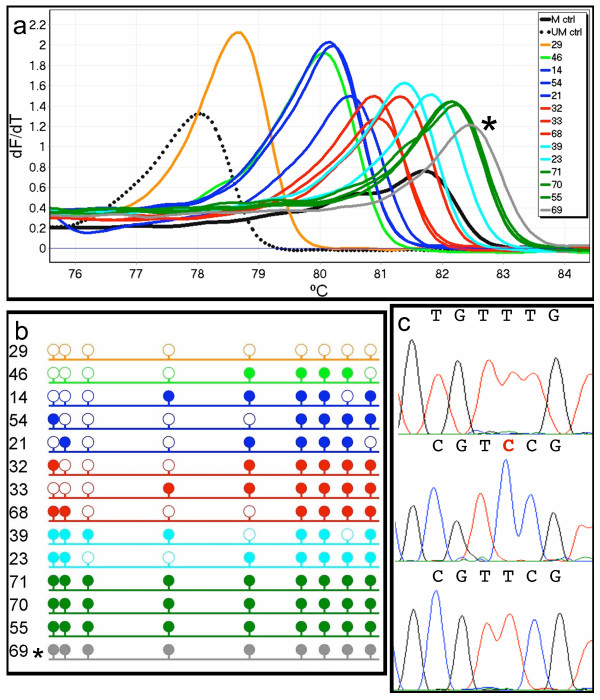
**Sequencing analysis of dMS-HRM products from an acute myeloid leukaemia sample**. PCR clones from sample 9164 were selected for sequencing as it showed the most variation, and was therefore the best example of the relationship of peak position to the degree of methylation. One representative of each peak observed from dMS-HRM is shown (a). Peaks are grouped within colours according to the number of CpG dinucleotides methylated as shown by sequencing and represented by lollipops, where open and filled circles represent unmethylated and methylated CpG sites, respectively (b). The order of clones in panel (b) reflects the order of peaks from left to right. The asterisk (*) marks PCR clone 69, as although it showed complete methylation, it also contained an unconverted cytosine. This is shown in the sequencing trace in the centre panel of (c), compared with an unmethylated sequence (above) and a methylated sequence (below). The incompletely converted cytosine is indicated in red. This incomplete conversion explains the higher Tm of this clone with respect to the other clones showing complete methylation.

Six AML samples that represented the range of variation observed were analysed digitally. Figure 3 shows the digital melting profiles of the selected AML samples. 4278 does not show any methylated alleles. 4383 and 4156 show products that correspond either to unmethylated products or have only few CpG sites methylated. 3224, 9164 and 730-06 cover a broad range of heterogeneously methylated alleles. Selected amplicons from one sample, 9164, were chosen for sequencing as it displayed the most variation in dMS-HRM products (Figure 4a). There is a close relationship between the Tm of the melting peaks and the number of CpG sites methylated, which vary only in the CpG sites being methylated.

In dMS-HRM, an increase in the melting temperature of the products relative to the controls can be seen (Figure 4a). However, sequencing of digital 'clones' obtained from the controls showed that their sequences were directly comparable to those from sample 9164 (Figure 4b). dMS-HRM analysis of control DNA was replicated with the template DNA being diluted into an equivalent amount of background DNA used for MS-HRM (fish sperm and unmodified genomic control DNA), and the same result was obtained (data not shown).

Incomplete conversion was detected on one occasion as the peak had a higher melting temperature than fully methylated 'clones' (Figure 4a). This was shown by direct sequencing to have been fully methylated and to have an additional unconverted cytosine (Figure 4c). Previous reports show that conversion of cytosine to uracil is 97.0 to 99.8% complete, whilst still leaving 5-methylcytosines intact [18,19]. Such a low non-conversion rate is not expected to be a major problem for dMS-HRM.

## Discussion

In our work on developing MS-HRM [10], we found that many promoter regions were heterogeneously methylated in cancer specimens (Candiloro et al, unpublished results). Heterogeneously methylated DNA samples result in multiple products after PCR. Not only will there be many different homoduplexes, but multiple heteroduplexes will also form between similarly methylated sequences, giving rise to distinctive melting curves. These often do not lie within the range defined by fully methylated and unmethylated DNA controls due to the earlier melting of heteroduplexes relative to homoduplexes. Thus MS-HRM allows ready identification of heterogeneously methylated samples (Figure 2). Depending on the amount of DNA methylation (and the amount of normal cells present in the tumour sample), the resulting melting curve is flattened and exhibits a complex melting pattern.

Quantification of the degree of heterogeneous methylation by MS-HRM remains problematic. MS-HRM shows great sensitivity when the samples contains a mixture of fully methylated and fully unmethylated templates [10] as it has been empirically observed that the sequence differences between these are too great for heteroduplexes to form. However, the sensitivity of MS-HRM is considerably diminished for heterogeneously methylated samples due to multiple heteroduplex formation creating a melting profile that cannot be directly compared with methylated and unmethylated controls.

To use a PCR-based technique, PCR bias needs to be eliminated or at least minimised. Given that the DNA methylation pattern in the entire interrogated sequence, and not just the primer binding sites, influences PCR bias [20], eliminating bias in a heterogeneous population of alleles becomes nearly impossible. It must also be kept in mind that cloning from a heterogeneous pool may introduce a cloning bias [21,22].

Digital PCR from single templates eliminates these problems [23]. When single molecules are amplified, there is no bias as there is no competition between different molecules within a single reaction.

We previously introduced dMS-HRM as a tool to count the number of methylated and unmethylated alleles for the *BRCA1 *promoter [11]. In this communication, we have adapted this methodology to examine the complexity and the degree of methylation of the *CDKN2B *promoter. Cameron et al determined that about 40% of the CpG sites within the *CDKN2B *CpG island need to be methylated to achieve complete silencing of the *CDKN2B *gene, regardless of the exact CpG methylation pattern [4]. Detection of DNA methylation at the *CDKN2B *promoter has been problematic as many methodologies are not well suited to the study of heterogeneously methylated regions.

A cost- and time-effective quantitative method is desirable for analysis of both research and clinical specimens where it is useful to know both the number of methylated templates and the degree of their methylation. Bisulphite sequencing of individual clones is an effective research approach but is too laborious and expensive for routine clinical use [4,5]. Our results show that dMS-HRM can be used as a more rapid alternative approach. In addition, dMS-HRM products may be directly sequenced. As dMS-HRM identifies the methylated templates, the need to sequence unmethylated templates is eliminated. Sequencing of the individual dMS-HRM amplicons enabled the identification of the exact methylation pattern. Sequencing confirmed that amplicons of similar Tm had similar numbers of methylated CpGs, although the actual positions varied (Figure 4).

MS-HRM and dMS-HRM can be compared with two other readily performed approaches that can recognise heterogeneous methylation. Denaturing gradient gel electrophoresis [6,24] is an effective way of visualising the complexity of heterogeneous methylation, but the methodology has not become widely used. Pyrosequencing is a comparatively rapid and quantitative method for methylation analysis [25-27]. Its quantitative approach and the characterisation of methylation pattern make it a useful method in a clinical setting, as shown for *CDKN2B *[7,28] and *MGMT *[29]. Heterogeneously methylated templates can be recognised if there is marked variation in the degree of methylation for the interrogated CpG positions. The great advantage of MS-HRM and dMS-HRM is that they are in-tube methodologies that are rapidly performed and do not need post-PCR processing as the melting analysis is carried out in the same reaction vessel as the amplification.

## Conclusion

MS-HRM can readily identify heterogeneously methylated templates by their characteristic melting patterns. This paper introduces dMS-HRM as an analytical method for the rapid study of heterogeneous methylation using the clinically important *CDKN2B *(*p15*) gene as an example. Although MS-HRM will often be sufficient to assess the methylation status of the *CDKN2B *gene, dMS-HRM will provide a more readily interpretable visualisation which may be quantified by scoring the individual peaks. dMS-HRM can also rapidly generate clonal templates for sequencing or pyrosequencing, eliminating the need for conventional cloning. Moreover, dMS-HRM will significantly reduce the amount of sequencing required as only clearly methylated amplicons will need to be sequenced.

## List of abbreviations used

AML: acute myeloid leukaemia; dMS-HRM: digital methylation-sensitive high resolution melting; MS-HRM: methylation-sensitive high resolution melting; WGA: whole genome amplification.

## Competing interests

The authors declare that no competing interests exist. The Peter MacCallum Cancer Centre and the University of Aarhus have a provisional patent on the MS-HRM methodology.

## Authors' contributions

ILMC participated in experimental design, performed the experiments and data analysis and co-wrote the manuscript. TM participated in experimental design, performed the data analysis and co-wrote the manuscript. PH provided the acute myeloid leukaemia samples. AD conceptualised the study, participated in experimental design, supervised the work and co-wrote the manuscript. All authors contributed to the writing of the manuscript and have read and approved the manuscript.
